# Discriminant analysis of farmers adoption of improved maize varieties in Wa Municipality, Upper West Region of Ghana

**DOI:** 10.1186/s40064-016-3196-z

**Published:** 2016-09-08

**Authors:** Abukari Alhassan, Hussein Salifu, Atinuke O. Adebanji

**Affiliations:** 1 Department of Statistics, Faculty of Mathematical Sciences, University for Development Studies, P.O. Box 24, Navrongo, U/E Region Ghana; 2 Department of Mathematics, Kwame Nkrumah University of Science and Technology, Kumasi, Ghana

**Keywords:** Varietal attributes, Adoption, Discriminant analysis, Quadratic classification

## Abstract

This study employed the quadratic classification function analysis to examine the influence of farmer’s socio-demographic and varietal characteristics of maize on adoption of improved maize varieties (IMVs) in the Wa Municipality of the Upper West region of Ghana. The results showed that, farm labour, information availability about the variety, weed resistance, low yielding variety, early maturity and water stress resistance are the major discriminating variables in classifying farmers in the Municipality. The study however revealed that maize experience, low yield, information availability and cost of variety were the most influential discriminating variables between adopters and non-adopters of IMVs. The study recommended the need to improve on the level of farmers’ education, ensure steady access to extension services and improvement in varietal characteristics identified in the study.

## Background

Maize is an adaptable crop, growing across a broad range of agro ecological zones. In Ghana, maize is a major source of carbohydrates and it is cultivated mostly in the southern regions, upper west, upper east and northern region of Ghana.

Maize also determines a household food security such that a low-income household is considered food insecured if it has no maize stock, regardless of other foods the household has at its disposal (Tweneboah 2000).

Ghana’s maize export levels have increased over the years from 2000 to 5000 metric tons from 2012 to 2016. Current yield of maize in Ghana stands at 1 metric ton per hectare (www.indexmundi.com/agriculture/ghana). Worldwide production of maize is 785 million tons, with Africa producing 6.5 % with the largest African producer being Nigeria with nearly 8 million tons, followed by South Africa. Africa imports about 28 % of the required maize from countries outside the continent (IITA 2016).

The current levels of maize yield suggest that Ghana is still not self-sufficient in maize production. Some experts have attributed it to low adoption of productivity-enhancing technologies, including improved varieties and management practices.

However, output variability is a major source of production risk under subsistence agriculture, especially when production depends solely on rainfall. Output variability affects both marginal gains and total farm output that influence food security at the household level. Food security is the most important priority for most subsistence farmers. Farmers prefer improved maize seeds that are stable in yield at different level of moisture availability (Moshi et al. 1990). Farmers avoid improved maize seeds that are highly variable in terms of yield as they pose food insecurity to households. The plan for reduction of yield variability will therefore influence which variety to adopt or not to adopt. At the household level, adoption choices are then formulated based on socioeconomic circumstances faced by the farmer and the attributes of the technology (CIMMYT 1993).

Ragasa et al. (2013), reported that, adoption of improved maize varieties (IMVs) have not seemed to increase since the 1997 survey of adoption. Out of the total maize area, 61 % was planted with modern varieties while only 15 % was planted with certified seeds. The research systems in the country have been very active in developing and releasing new varieties. They further intimated that, a 1992 variety, Obatanpa, is still the predominant variety and has gained popularity over the years than the newer varieties. This rather very high weighted-average varietal age (23 years) in Ghana signals that either the research system produces many irrelevant varieties that are not solving farmers’ binding constraints or the agricultural extension system is unable to disseminate and educate farmers about the net benefits of new varieties.

Considerable literature exists in reporting attempts to explain the adoption of new IMVs using discriminant analysis (DA). Lakshman and Robert (1978) used DA to classify adoptors and Non adoptors of new variety of high yielding maize. Accessibility of resources was found to be a significant contributor to the adoption of high yielding maize. Luke et al. (2010) also used DA to investigate some factors that characterize farmers based on some starting conditions. Bashir and Wegrary (2014) studied the determinants of small holder farmers hybrid maize adoption in the drought prone Central Rift Valley of Ethiopia. They employed the linear discriminant function in their study to identify the significant socio-economic characteristics that discriminates among adoption of hybrid maize.

Thomson et al. (2014) used the logit model to model adoption of improved maize seed varieties in Southern Zambia. They also employed similar variables such as farmers age, maize farming experience, farm labour etc. Some other authors including Xiaolei et al. (2012) and Lee et al. (2007) have all developed classification procedures for selecting varieties of maize and maize hybrids respectively.

Based on the background literature, the determination of factors that contributes to adoption of IMVs in a population is imperative for the implementation of policy control measures as well as to improve livelihoods through sustainable increased productivity of maize.

The current study seeks to apply DA to classifying farmer’s adoption of IMVs in the Upper West Region of Ghana based on their socio-economic characteristics and varietal characteristics of maize.

## Methods

### Sampling procedure

This study involved a cross-sectional survey with 300 systematic sampled households growing maize in the two selected communities of Biihii and Kpongu in the Wa Municipality of Ghana. Biihii and Kpongu were selected purposely because of the importance of maize in the farming systems and the availability of maize technology dissemination programs in the two areas. Data was by means of a structured questionnaire, developed and used for gathering relevant information from the farmers. The instrument was administered to the respondents through a face-to-face interview of a convenient sample sizes of 135 households from Kpongu and 165 households from Beehii, with the assistance of the Savanna Agricultural Research Institute agents who interacted directly with the farmers at the local level.

### Background information of the study area

The Upper West Region (UWR) is typical Guinea savannah, with a high density of tree species. Broadly speaking, the low population densities have permitted a remarkable conservation of savannah vegetation, quite unlike much of the remainder of Northern Ghana. The UWR forms part of savannah accelerated development authority (SADA) zone. The climatic regime is semi-arid with annual rainfall ranging between 700 and 1200 mm. The rain falls in a 7-month season from April to October (Ghana Statistical Service 2010).

**Fig. 1:**
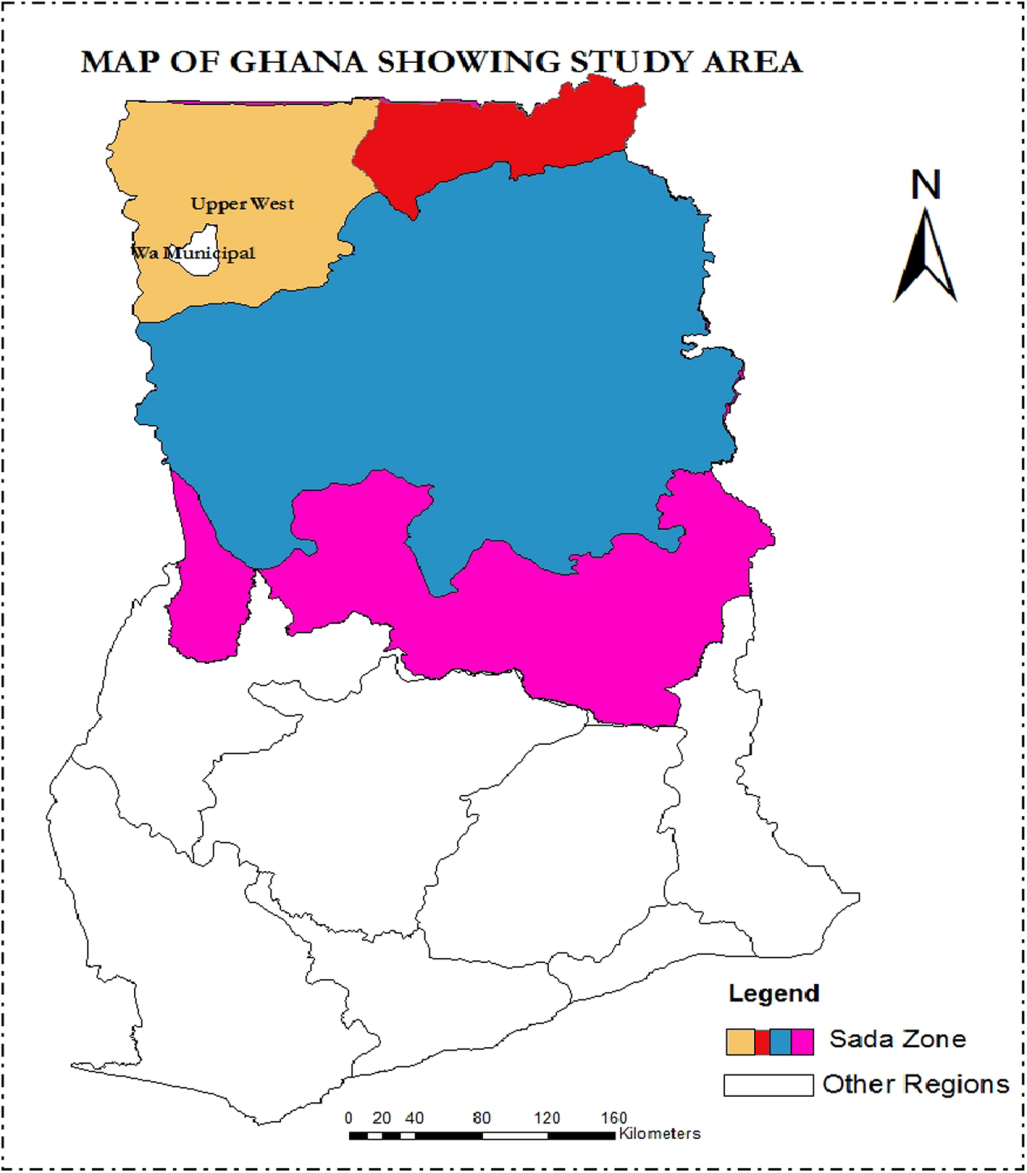
Map of Ghana showing the study area

The Wa Municipality is one of the nine administrative areas (District Assemblies) that make up the Upper West Region (UWR) of Ghana. The Municipality lies within latitudes 1°40″N–2°45″N and longitudes 9°32″–10°20″W (Ghana Statistical Service 2010).Wa Municipality has a total population of 702,110. Wa town alone has a population size of 107,214 constituting 15.3 % of the region’s population. The growth rate of the Municipality varies between 2.7 % for rural and 4 % for the urban. Majority of the households (30.9 %) in the municipality are engaged in agriculture, with almost 82.9 % of these agricultural households involved in crop farming (Ghana Statistical Service 2010). Figure 1 shows the map of Ghana indicating the study area.

### Discriminant analysis

Discriminant analysis is a multivariate statistical technique used to determine which variables discriminate between two or more naturally occurring groups. Through DA, one may classify farmers into two or more mutually exclusive and exhaustive groups on the basis of a set of independent variables.

### Linear discriminant/classification model $(\Sigma_{i} = \Sigma_{j} = \Sigma )$

Supposes the two population’s π_1_ and π_2_ has multivariate normal densities $X^{\prime } = [x_{1}, x_{2} ,\ldots,x_{p} ]$ with mean vectors and covariance matrices, $\mu_{1}$, $\Sigma_{1}$ and $\mu_{2}$, $\Sigma_{2}$ respectively given by

1 $$f_{i} (x) = \frac{1}{{(2\pi )^{p/2} \left| \Sigma \right|^{1/2} }}\exp \left[ { - \frac{1}{2}(x - \mu_{i} )^{\prime } \Sigma^{ - 1} (x - \mu_{i} )} \right]\quad {\text{for}}\;i = 1,2.$$

The allocation rule that minimizes the expected cost of misclassification (ECM) is given by:

Allocate $x_{0}$ to $\pi_{1}$ if

2 $$(\mu_{1} - \mu_{2} )^{\prime } \Sigma^{ - 1} x_{0} - \frac{1}{2}(\mu_{1} - \mu_{2} )^{\prime } \Sigma^{ - 1} (\mu_{1} + \mu_{2} ) \ge \ln \left[ {\left( {\frac{c(1/2)}{c(2/1}} \right)\left( {\frac{{p_{2} }}{{p_{1} }}} \right)} \right]$$

Allocate $x_{0}$ to $\pi_{2}$ otherwise (Johnson and Wichern 2007).

The population parameters in Eq. (2) can be replaced by its sample estimates; $\bar{x}_{1} ,\bar{x}_{2}$ and $S_{pooled}$. Given a special case when there are equal prior probabilities and equal misclassification cost, then we assign $x_{0}$ to $\pi_{1}$ if:

3 $$(\bar{x}_{1} - \bar{x}_{2} )^{\prime } S_{pooled}^{ - 1} x_{{}} - \frac{1}{2}(\bar{x}_{1} - \bar{x}_{2} )^{\prime } S_{pooled}^{ - 1} (\bar{x}_{1} + \bar{x}_{2} )$$

### The quadratic classification model ($\Sigma_{i} \ne \Sigma_{j} )$

The decision boundary or the minimum expected cost of misclassification is based on the density ratio ${{f_{1} (x)} \mathord{\left/ {\vphantom {{f_{1} (x)} {f_{2} (x)}}} \right. \kern-0pt} {f_{2} (x)}}$. Substituting multivariate normal densities with different covariance matrices into Eq. (1) after taking natural logarithms and simplifying, the resulting classification regions are:

$$R_{1} : - \frac{1}{2}x^{\prime } \left( {\Sigma_{1}^{ - 1} - \Sigma_{2}^{ - 1} } \right)x + \left( {\mu_{1}^{\prime } \Sigma_{1}^{ - 1} - \mu_{2}^{\prime } \Sigma_{2}^{ - 1} } \right)x - K \ge \ln \left[ {\left( {\frac{c(1/2)}{c(2/1)}} \right)\left( {\frac{{p_{2} }}{{p_{1} }}} \right)} \right]$$

4 $$R_{2} : - \frac{1}{2}x^{\prime } \left( {\Sigma_{1}^{ - 1} - \Sigma_{2}^{ - 1} } \right)x + \left( {\mu_{1}^{\prime } \Sigma_{1}^{ - 1} - \mu_{2}^{\prime } \Sigma_{2}^{ - 1} } \right)x - K \ge \ln \left[ {\left( {\frac{c(1/2)}{c(2/1)}} \right)\left( {\frac{{p_{2} }}{{p_{1} }}} \right)} \right]$$

The allocation rule that minimizes the expected cost of misclassification is given by replacing the population parameters with sample estimates, the minimum ECM then becomes:

Allocate $x_{0}$ to $\pi_{1}$ if

5 $$- \frac{1}{2}x_{0}^{\prime } \left( {S_{1}^{ - 1} - S_{2}^{ - 1} } \right)x_{0} + \left( {\bar{x}_{1}^{\prime } S_{1}^{ - 1} - \bar{x}_{2}^{\prime } S_{2}^{ - 1} } \right)x_{0} - K \ge \ln \left[ {\left( {\frac{c(1|2)}{c(2|1)}} \right)\left( {\frac{{P_{2} }}{{P_{1} }}} \right)} \right]$$

Allocate $x_{0}$ to $\pi_{2}$ otherwise (Johnson and Wichern 2007).

Where

6 $$K = \frac{1}{2}\ln \left( {\frac{{|\Sigma_{1} |}}{{|\Sigma_{2} |}}} \right) + \frac{1}{2}\left( {\bar{x}_{1}^{\prime } S_{1}^{ - 1} \bar{x}_{1} - \bar{x}_{2}^{\prime } S_{2}^{ - 1} \bar{x}_{2} } \right)$$

$\left( {\frac{c(1|2)}{c(2|1)}} \right)$ is the expected cost ratio and $\left( {\frac{{P_{2} }}{{P_{1} }}} \right)$ is the prior probability ratio.

We assume that for each of the populations there are equal prior probabilities and equal misclassification cost, then the allocation rule reduces to

7 $$- \frac{1}{2}x_{0}^{\prime } \left( {S_{1}^{ - 1} - S_{2}^{ - 1} } \right)x_{0} + \left( {\bar{x}_{1}^{\prime } S_{1}^{ - 1} - \bar{x}_{2}^{\prime } S_{2}^{ - 1} } \right)x_{0} - K \ge 1$$

### Error rate estimation

The holdout procedure sometimes referred to as jackknifing or cross-validation was used to evaluate the performance of the classification function. This method usually holds one observation at a time and classifies the hold out observation. This process is repeated until all observations are classified which produced almost unbiased estimates of the misclassification probabilities (Lachenbruch and Mickey 1968).

### Organization of data

Adoption of IMVs was defined by two categories; adopters and non-adopters. The different categories of adoption were conceptually interpreted accordingly as follows: non-adopters means the farmer use local maize variety whiles adopters referred to farmers who used IMV.

The independent variables used in the study were some attributes of the improved varieties which were explored using a 5-points Likert scaled with 1 = No effect, 2 = Little effect, 3 = Not sure, 4 = Moderate effect, 5 = Extreme effect. These variables were as follows: storage/streak resistance, recycle grain (replanting), mature late, weed resistance, grain quality (grain colour/texture), low yield, water stress resistance, mature early, fertilizer requirement, information availability, diseases/pest resistances, soil fertility requirement and cost. In addition to these variables, farmers characteristics such as age of farmer, total farm labour and experience in maize farming were used in the analysis.

## Results and discussion

The descriptive analyses of farmer’s socio-demographic characteristics results in Table 1 revealed a mean age of 41 years with a standard deviation of 13.38 for farmers who are adopters of IMVs and a mean age of 39 years and a low standard deviation of 8.59 for non-adopters. With respect to mean farm labour, the result did not reveal much variation between the different categories of adoption. The results also showed higher years (11) of maize farming experience for adopters of IMVs as compared to non-adopters (9 years) of IMVs.

**Table 1:** Descriptive statistics of some selected variables. *Source*: analysis from survey data in Beehi and Kpongu

Variables	Adopters		Non-adopters	
	Mean	SD	Mean	SD
Farmer’s age	41.44	13.38	38.75	8.59
Farm labour	5.41	3.34	4.73	2.30
Maize experience	11.12	4.02	9.13	3.19

Seven (7) out of twenty-seven approved IMVs were found to be cultivated in the study areas. IMVs such as Obatanpa, Mamaba and Aburohemaa (34, 20.7 and 16.3 %) respectively were most popular. This may be as a result of the fact that, these varieties have been introduced quite a long time ago. The remaining varieties are quite new in the system hence its low patronage.

In order to investigate the determinants of adoption of IMVs, the Box M test of equality of population covariance matrices of the two groups of adoption under study was first tested. The log determinant of the groups was found as shown in Table 2. Under the null hypothesis of equal covariance matrices, the Box M test was significant at 1 % level, indicating a violation of the assumption of equal covariance matrices.

**Table 2:** Test for equality of population covariance matrices. *Source*: analysis from survey data in Beehi and Kpongu

Adoption	Rank	Log determinant	Chi square	df	P value
Non-adopters	9	−159.91941	6033.3719	190	0.0001*
Adopters	19	1.9237			
Pooled	19	3.80536			

* Significant at 1 %

A quadratic classification function was then fitted to the data. Results of the quadratic classifier showed a significant performance at 1 % significant level under the respective multivariate test statistics (Table 3). The test for differences in the mean vectors ($\mu_{1}$ and $\mu_{2}$) is viewed as test for significance of the separation that is achieved. The QDF derived to classify farmers into their respective groups under unequal prior probability (Table 4) and equal misclassification cost has been determined and written as:

**Table 3:** Test of model adequacy. *Source*: analysis from survey data in Beehi and Kpongu

Test statistic	Value	F-value	DF1	DF2	P value
Wilks’ Lambda	0.4942	14.98	19	278	0.0001*
Pilla’s Trace	0.5058	14.98	19	278	0.0001*
Hotelling Lawley Trace	1.0235	14.98	19	278	0.0001*
Roy’s Greatest Rooa	1.0235	14.98	19	278	0.0001*

* Significant at 1 %

**Table 4:** Quadratic function classification results. *Source*: analysis from survey data in Beehi and Kpongu

	Classified		
	Non-adopters	Adopters	Total
*True/original*			
Non-adopters	42	0	42
Percent	100	0	100
Adopters	3	255	258
Percent	1.16	98.84	100
Total	45	255	300
Percent	15	85	100
Error rate	0	0.0116	0.01
Priors	0.14	0.86	
*Cross*-*validation*			
Non-adopters	41	1	42
Percent	97.62	2.38	100
Adopters	3	255	258
Percent	1.16	98.84	100
Total	44	256	300
Percent	0.14	85.33	100
Error rate	0.238	0.0116	0.0133

8 $$(x - \bar{x}_{1} )S_{1}^{ - 1} (x - \bar{x}_{1} ) - (x - \bar{x}_{2} )S_{2}^{ - 1} (x - \bar{x}_{2} ) \ge 1.815$$

Table 4 presents the result of classification and misclassification rates. 98.84 % of the farmers were correctly classified as adopters of IMVs with a misclassification rate of 1.16 into the non-adopters group. However, none of the non-adopters of IMVs were misclassified and a 100 % correct classification was achieved. Consequently, an overall error rate of 0.01 was achieved under the classification model. Further, the cross validation option provides a better assessment of classification accuracy. This classification is also done for each observation; however, the discriminant function used in each case was constructed by taking that observation out of the data set. Thus, every data point was reclassified as if it were a new unknown observation. This provided a more Conservative accuracy assessment. For this data, adopters of IMV’s now showed an error rate of 1.16 % while non-adopters of IMV’s were 2.38 %. Overall, 13.3 % of the observations were misclassified under the cross validation. The results also indicated that, in all approximately 99.0 % (1–0.01) correct classification of farmers was achieved under classification with QDF as well as 98.67 % (1–0.0133) correct classification rate under the cross validated results.

Another way of evaluating the performance of the discriminant function is to investigate the eigenvalue and the canonical correlation coefficient. The ability of a discriminant function to separate groups can be judged from the magnitude of the canonical correlation. If the total structure coefficient is equal to or greater than 0.30 it is considered useful (Johnson and Wichern 2007). The eigenvalue and the canonical correlation coefficient further affirmed that the model was correctly specified. The hypothesis that the canonical correlation in the current row and all that follows are zero was rejected at 5 % significant level which further affirmed that QDF was correctly specified (See Table 5).

**Table 5:** Test of canonical correlation. *Source*: analysis from survey data in Beehi and Kpongu

	Can. corr.	Adjt. can. corr.	Approx. SE	Square can. corr.	Eigenvalue
Function 1	0.711205	0.69074	0.028676	0.505812	1.0235

Test	Likelihood ratio	F-value	df	df	P value
Function 1	0.49418785	14.98	19	278	0.0001*

*Can. corr.* canonical correlation

* Significant at 5 %

**Fig. 2:**
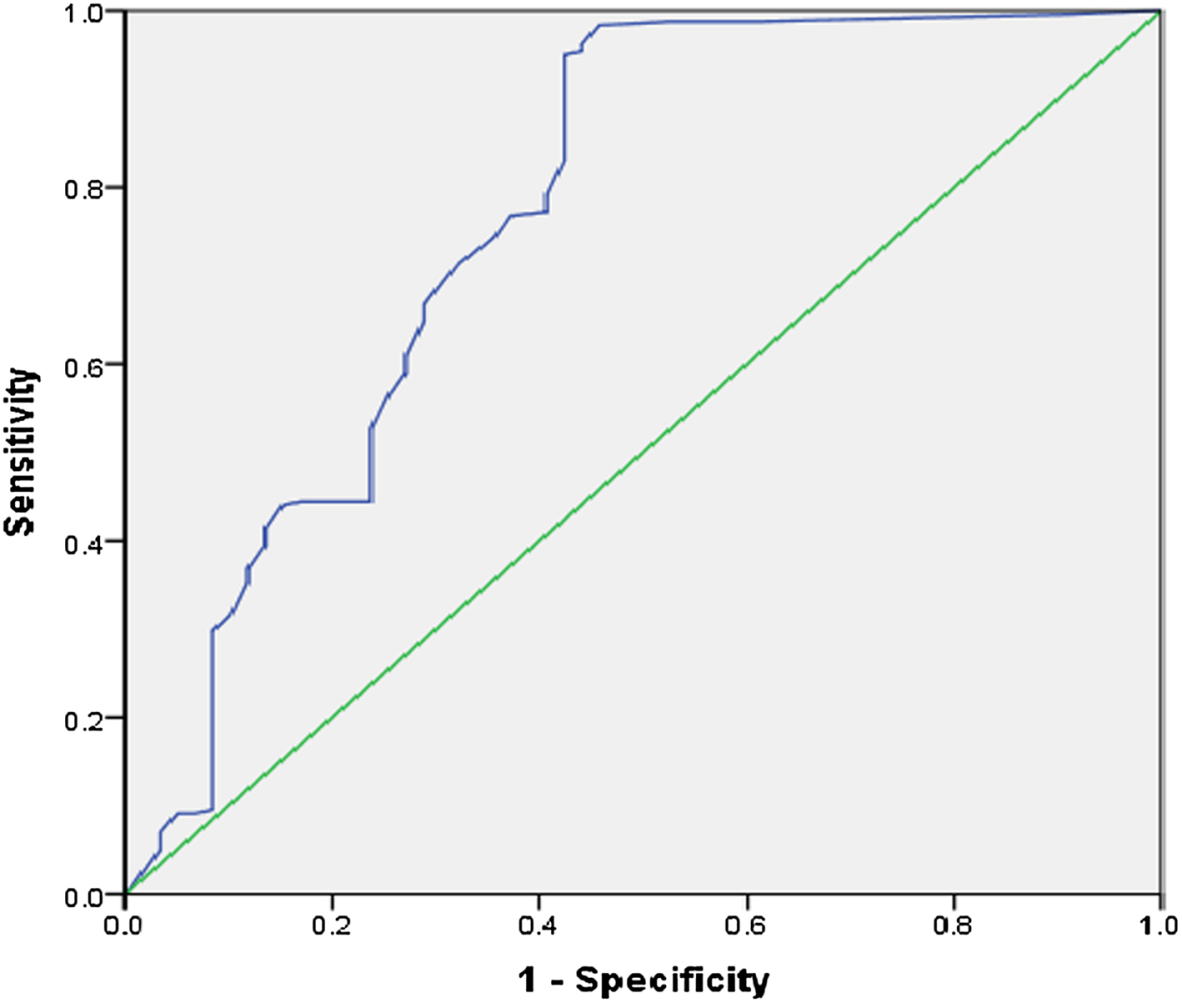
ROC curve

The performance of the model was further investigated using the receiver operating characteristic (ROC) curve (Fig. 2). The results showed a large Area Under the Curve (AUC) of 76.8 % and significant *P* value at 5 % significant level which further affirmed that, the model was correctly specified. Also, the Tukey’s test of additivity was significant (*F*-Value = 15.068 and *P* Value = 0.000) at the 5 % level of significance indicating that, there is no multiplicative interaction among the items of the likert scale affirming the independence of the variables.

To identify the minimum number of variables that is important for discrimination and their level of significance in contributing to discrimination, the univariate test of class means was used (Table 6).The results indicated that, maize farming experience, variety availability, late maturing, weed resistance, low yield, fertilizer requirements, information availability and cost of variety was significant at 1 % (*P* < 0.01). While total farm labour, early maturity, and disease/pest resistance were significant at 5 % (*P* < 0.05). The results of the study contradicted earlier studies (Ebojei et al. 2012; Bashir and Wegrary 2014) which reported significant influence of farmer’s age and farm size on adoption of improved seeds. However, the results confirmed earlier results of Thomson et al. (2014) and Cavane (2009) which indicated that, adoption of IMVs was influenced by expected yields, attitudes toward production trait of IMVs and knowledge (information availability).

**Table 6:** Univariate test of class means. *Source*: analysis from survey data

Variables	Total SD	R-square	Adjusted R-square	F-value	P value
Age of farmer’s	9.7611	0.0064	0.0064	1.89	0.1705
Total farm labour*	2.5576	0.0147	0.0149	4.48	0.0367
Maize experience**	3.4623	0.1663	0.0195	59.04	0.0001
High yield	0.8418	0.0070	0.0070	2.06	0.1502
Availability**	1.2566	0.0334	0.0346	10.23	0.0015
Storage/streek resistance	1.0111	0.0001	0.0001	0.02	0.8791
Re-propagation	0.9376	0.0000	0.0000	0.000	0.9630
Late maturity**	1.2979	0.0224	0.0230	6.80	0.0096
Weed resistance**	0.7878	0.0350	0.0362	10.72	0.0012
Grain quality	1.1945	0.0053	0.0054	1.59	0.2089
Low yield**	1.5180	0.0839	0.0916	27.12	0.0001
Water stress resistance	1.2045	0.0008	0.0008	0.24	0.6277
Nutritional value	1.2492	0.0000	0.0000	0.01	0.9183
Early maturity*	1.1876	0.0203	0.0207	6.13	0.0138
Fertilizer requirement**	1.3025	0.0464	0.0487	14.41	0.0002
Information availability**	1.1023	0.0619	0.0717	21.24	0.0001
Disease/pest resistance*	1.0839	0.0141	0.0143	4.24	0.0404
Soil fertility requirement	0.9901	0.0011	0.0011	0.32	0.5735
Cost of variety**	1.0399	0.0770	0.0835	24.71	0.0001

** Significant at 1 %; * significant at 5 % level

The amount of variation explained by each discriminating variable is provided by the R-square which is adjusted for bias. The results show (See Table 6) that low yield, information availability and cost of variety explained large proportions of the variability (9.16 %, 7.17 % and 8.35 %) among the classes and hence indicates the level of contribution of these variables to group separation.

The structure matrix in Table 7 provides another way of studying the importance of the variables in the discriminant function. The ability of a discriminant function to separate groups can be judged from the magnitude of the canonical correlation. The results showed that, maize farming experience, low yield variety, fertilizer requirement, information availability and cost of variety are important discriminating variables. Thus a farmer with these attributes is more likely to adopt IMVs in the Wa municipal area.

**Table 7:** Structure matrix. *Source*: analysis from survey data in Beehi and Kpongu

Variables	Function
Age of farmer’s	0.111919
Total farm labour	0.170265
Maize experience	0.573367
High yield	−0.117490
Availability	0.256953
Storage/streak resistance	−0.012442
Re-propagation	−0.003792
Late maturity	0.210645
Weed resistance	−0.262888
Grain quality	0.102637
low yield	0.407332
Water stress resistance	−0.039658
Nutritional value	0.008392
Early maturity	−0.200340
Fertilizer requirement	0.302978
Information availability	0.363782
Disease/pest resistance	0.167080
Soil fertility requirement	0.040035
Cost of variety	0.390268

It is also evident from the result (Table 7) that, maize farming experience, low yield, information availability and cost of variety has relative high coefficient value of 0.57, 0.41, 0.36 and 0.39 respectively, and hence has a significant influence on discriminating between adopters and non-adopters of IMVs. This implies that, the more farmers are informed about IMVs the more likely they adopt the variety. Also, the results revealed that a more experienced farmer has a greater probability of adopting IMVs. On the other hand, the lower the yields of IMVs, the less likely farmers’ adopt them. However, Hussein et al. (2015) reported negative influence of low yield and information availability on adoption of IMVs which contradicts the results of the current study. The results of this study supports the recommendations of Feder et al. (1985), Feder and Umali (1993) that, farmer perceptions of technology-specific characteristics significantly condition technology adoption decisions.

Table 8 presents standardized and unstandardized canonical discriminant coefficients of the QDF with class means of 2.46 and −0.41 respectively for non-adopters and adopters of IMVs. Future observations of farmers can be classified by evaluating the unstandardized canonical function. Farmers whose canonical coefficient is closer to the class means are classified as belonging to that class. The generalized squared distance function of the analysis is given as:

**Table 8:** Unstandardized and standardized canonical discriminant coefficient. *Source*: analysis from survey data in Beehi and Kpongu

Variables	Unstandardized	Standardized
Age of farmer’s	−0.0019	−0.0187
Total farm labour	−0.0431	−0.1102
Maize experience	0.2049	0.7093
High yield	0.0278	0.0234
Availability	0.1097	0.1378
Storage/streak resistance	0.1381	0.1396
Re-propagation	−0.1134	−0.1063
Late maturity	0.0662	0.0859
Weed resistance	−0.6249	−0.4924
Grain quality	−0.1567	−0.1872
Low yield	0.4759	0.7224
Water stress resistance	0.2399	0.2889
Nutritional value	−0.0589	−0.0736
Early maturity	−0.1579	−0.1875
Fertilizer requirement	0.1227	0.1599
Information availability	0.8568	0.9445
Disease/pest resistance	−0.1497	−0.1622
Soil fertility requirement	−0.2946	−0.2917
Cost of variety	0.3677	0.3824

9 $$D_{j}^{2} (x) = (x - \bar{x}_{(x)j} )^{\prime } S_{(x)j}^{ - 1} (x - \bar{x}_{(x)j} ) + \ln \left| {S_{(x)j} } \right|$$

The results of this study confirmed earlier research which indicated that adoption of improved maize technologies, was influenced by attitude toward varietal traits, knowledge to apply the technology, and the role of extension services in dissemination of improved technology (Kaliba et al. 2000; Abebaw and Belay 2001; Hintze et al. 2003; Gwary et al. 2012).

## Conclusions and recommendations

In this study, the determinants of adoption of IMVs in the Wa Municipality of the Upper West Region of Ghana were investigated. The results show that farm labour, maize experience, availability of variety, information availability, low yield, early maturity, fertilizer requirement and cost of variety were the major discriminating variables in classifying farmers in the study areas. The study revealed that maize experience, low yield, information availability and cost of variety were the most influential discriminating variables between adopters and non-adopters of IMVs. It is therefore recommended that soil scientists and crop breeders should consider an improvement in the specific varietal characteristics identified as influencing adoption of IMVs. Priority can be given to development of maize varieties whose fertilizer requirement is low and which are early maturing and high yield. Also agricultural extension division of the Ministry of Food and Agriculture (MOFA) should consider organizing on-farm trials with the farmers in order to accelerate their adoption of the IMVs. The ministry of Agriculture through the public information department should intensify public educations through radio, community dabbers and any available platform to increase farmer’s knowledge on new agricultural technologies.
